# Supplementary material for: Public support for global vaccine sharing in the COVID-19 pandemic: Evidence from Germany
Source: PLoS One. 2022 Dec 14;17(12):e0278337. doi: 10.1371/journal.pone.0278337 (PMC9750013; doi:10.1371/journal.pone.0278337)
Supplement: S1 File — (PDF) [file pone.0278337.s001.pdf]

Pre-analysis plan: German vaccine attitude studies

9/10/2021

Contents

Study 4.2: Solidarity experiment **1**
Assignment . . . 2
Outcomes . . . 2
Estimands and Analysis . . . 2
Design declaration . . . 3

Study 4.2: Solidarity experiment

We implement a $2 \times 2 \times 5$ factorial design.

We lead with introductory text:

> The following is about vaccination progress in Germany and the rest of the world.
>
> The vaccination campaign against the coronavirus in Germany is now well advanced and anyone who wanted to be vaccinated could do so. The benefits of a third booster vaccination are currently being discussed. In contrast, many other, poorer countries are still at the very beginning with vaccinations and almost no one there has received a single vaccination yet.
>
> A total of around 11 billion vaccine doses are needed to vaccinate all people around the world who are ready to vaccinate. Although the production of vaccines is in full swing, there is currently not enough vaccine available to carry out the first and second vaccinations in the poorer countries and the third vaccination in the richer countries at the same time
>
> Variations are then as follows:

Z1: Trading importance

- 0 Control: It has no negative impact on the German economy if there are no vaccinations in poorer countries.
- 1 Treatment: The German economy shrinks by around 5% if there are no vaccinations in poorer countries.

Z2: Risk

- 0 Control: The risk of new mutations of the coronavirus does not increase noticeably in Germany if there are no vaccinations in poorer countries.
- 1 Treatment: The risk of new mutations of the coronavirus increases considerably in Germany if there are no vaccinations in poorer countries.

Z3: Deal

- 0 Control: There is no international deal on the global distribution of vaccines to poorer countries.
- 1 Treatment: There is an international deal on the global distribution of vaccines to poorer countries. 20 other countries are involved in the agreement, which together contribute a total of 20 billion euros.
- 2 Treatment: There is an international deal on the global distribution of vaccines to poorer countries. 40 other countries are involved in the agreement, which together contribute a total of 20 billion euros.

- 3 Treatment: There is an international deal on the global distribution of vaccines to poorer countries. 20 other countries are involved in the agreement, which together contribute a total of 40 billion euros.
- 4 There is an international deal on the global distribution of vaccines to poorer countries. 40 other countries are involved in the agreement, which together contribute a total of 40 billion euros.

Note that $Z3$ can itself be interpreted as a $2 * 2 + 1$ sub design that can be coded into:

- Z4: 0, 20, 40 other countries
- Z5: 0, 20, 40 billion provided by others

Assignment

Each subject sees two conditions, producing a total of $20 \times 19 = 380$ versions, assigned independently.

Outcomes

Subjects are asked about amounts of vaccines that Germany should share and financial contributions Germany should make in each condition.

Outcome 1:

> The total costs to meet global vaccination needs amount to around 70 billion euros. A contribution from Germany of one billion euros to this fund would cost the population in Germany the equivalent of around 12 euros per person.
>
> For each of the two scenarios, please indicate how many euros Germany should contribute to this fund for global vaccination. (Options range from 0 to 70 billion; Millions can be specified, separated by commas (e.g. 0.1 billion for 100 million))

- Vignette 1 ..billion Euro
- Vignette 2 ..billion Euro

Outcome 2:

> Germany will still have around 200 million vaccine doses available by the end of the year. If Germany wanted to offer all of its citizens a third vaccination, Germany would have to reserve around 70 million of these vaccine doses.
>
> For each of the two scenarios, please indicate what proportion of these 200 million vaccine doses Germany should contribute to the global distribution of vaccine doses to poorer countries. (Options range from 0 to 200m)

- Vignette 1 ..million doses
- Vignette 2 ..million doses

Estimands and Analysis

Our primary analysis examines:

(a) baseline support for contributions, supoprt for contributions under future conditions
(b) the effects of economic and health risks on contributions costs
(c) the effects of the numbers of others contributing
(d) the effects of amounts others are contributing

The primary analysis is implemented by demeaning treatments and estimating a saturated model:

```
lm_robust(DY = Z1*Z2*Z4*Z5, fixed_effects = ~id)
```

A secondary analysis will seek to estimate a model in which citizens place a value on own contributions x_i, given contributions by others $n\overline{x}_{-i}$:

- total contributions (varied by Z5), moderated by risks (varied by Z1 and Z2)

- costs of own contributions
- costs of deviation from a norm of giving in line with contributions by others (mean contributions depend on Z4 and Z5)

$$(\alpha_i + \beta_i Z_1 + \gamma_i Z_2) \log \left(\sum_j x_j \right) - x_i^2 - \gamma(x_i - \kappa \overline{x}_{-i})^2$$

These analyses are supplemented using heterogeneity analysis to assess dependence on (a) party support and (b) migration background. An exploratory causal forests analysis to identify sources of heterogeneity across respondents.

Design declaration

A simple declaration captures the main features of this design with analysis 1 implemented using first differences. The structural model is not integrated here.

```
N = 7500

settings <-
  expand_grid(Z1_1 = 0:1, Z1_2 = 0:1,
              Z2_1 = 0:1, Z2_2 = 0:1,
              Z3_1 = 0:4, Z3_2 = 0:4,
              ) %>%
  filter(!((Z1_1==Z1_2) &  (Z2_1==Z2_2) & (Z3_1==Z3_2)))

b <- .05

design <-

  declare_model(
    N = N,
    U = rnorm(N),
    U_1 = rnorm(N),
    U_2 = rnorm(N),
    settings[sample(nrow(settings), N, replace = TRUE), ],
    Z4_1 = (Z3_1==1 | Z3_1==3) + (Z3_1==2 | Z3_1==4), # n countries
    Z5_1 = (Z3_1==1 | Z3_1==2) + (Z3_1==3 | Z3_1==4), # total $
    average_1 = ifelse(Z4_1 == 0, 0, Z5_1/Z4_1),
    Z4_2 = (Z3_2==1 | Z3_2==3) + (Z3_2==2 | Z3_2==4), # n countries
    Z5_2 = (Z3_2==1 | Z3_2==2) + (Z3_2==3 | Z3_2==4), # total $
    average_2 = ifelse(Z4_2 == 0, 0, Z5_2/Z4_2),
    Give_1 = U + U_1 + .2 + b*Z1_1 + .4*Z2_1 + .1*average_1 ,
    Give_2 = U + U_2 + .2 + b*Z1_2 + .4*Z2_2 + .1*average_2)  +
  declare_measurement(
    DY = Give_2 - Give_1,
    DZ1 = Z1_2 - Z1_1 - mean(Z1_2 - Z1_1),
    DZ2 = Z2_2 - Z2_1 - mean(Z2_2 - Z2_1),
    DZ3 = Z3_2 - Z3_1 - mean(Z3_2 - Z3_1),
    DZ4 = Z4_2 - Z4_1 - mean(Z4_2 - Z4_1)
    ) +
  declare_inquiry(ATE_1 = b) +
  declare_estimator(
    DY ~ DZ1*DZ2*DZ3*DZ4, model = lm_robust, term = "DZ1", inquiry = "ATE_1"
```

```
  )

df <- draw_data(design)
# lm_robust(Give_1 ~ Z1_1, data = df)
# lm_robust(Give_2 ~ Z1_2, data = df)
# lm_robust(DY ~ DZ1, data = df)

diagnose_design(design) %>%
  reshape_diagnosis() %>% kable(digits = 3)
```

Design	Inquiry	Estimator	Term	N Sims	Bias	RMSE	Power	Coverage	Mean Estimate	SD Estimate	Mean Se	Type S Rate	Mean Estimand
design	ATE_1	estimator	DZ1	500	0.00	0.03	0.48	0.95	0.05	0.03	0.03	0.00	0.05
					(0.00)	(0.00)	(0.02)	(0.01)	(0.00)	(0.00)	(0.00)	(0.00)	(0.00)
