# Supplementary material for: Public support for global vaccine sharing in the COVID-19 pandemic: Evidence from Germany
Source: PLoS One. 2022 Dec 14;17(12):e0278337. doi: 10.1371/journal.pone.0278337 (PMC9750013; doi:10.1371/journal.pone.0278337)
Supplement: S2 File — (PDF) [file pone.0278337.s002.pdf]

Pre-Analysis Plan: Solidarity: Evidence from the COVID-19 vaccination programme

29.04.2021

Contents

1 Sample

Our population of interest consists of all German citizens over the age of 18. We rely on a representative sample of 12,500 citizens across Germany which have already been questioned in one preceding panel wave in March 2021. In order to study the roll-out of the vaccine over time the project we will launch an online survey in Germany at the end of April 2021. We will draw on a sample of respondents with the help of the online-access panel provider Repondi. The questionnaire will take around 15 minutes.

2 Study Design

In the first experiment, participants will be randomly assigned to a treatment group which is exposed to a video explaining the benefits of global vaccine sharing and a control group which does not see a video. Subsequently respondents will be questioned about their willingness to donate money for global vaccine sharing. Afterwards all respondents will be included in a Conjoint experiment in which we randomize the following dimensions: absolute contribution to global vaccine sharing, contribution to global vaccine sharing relative to other countries, number of countries participating in global vaccine sharing, economic benefits and health benefits.

3 Pre-Treatment Covariates

Before both experiments, we collect a number of covariates that are predicted to moderate the effect of the treatments on our outcome variables. We include trust in government and local health system (Mesch & Schwirian, 2015), economic hardship and eduction (Bertoncello et al., 2020), minority group status and maternal age (Danis et al., 2010), risk perception (Brewer et al., 2007) and health status (Guay et al., 2019). Additionally, we collect data on political attitudes, attitudes towards migrants, and attitudes towards COVID-19 as well as indicators that identify groups who are eligable for the vaccine. Please see the enclosed questionnaire for details.

4 Survey Experiment 1: International solidarity

4.1 Motivation

In this work package, we seek to study the determinants of international solidarity among voters. We build on a growing body of liteature that investigated solidarity between European citizens in particular during the Eurozone crisis (Bechtel et al. 2017a; Kuhn et al. 2018; Kuhn et al. 2020; Stoeckel & Kuhn 2018) and citizen preferences for international climate agreements (Bechtel & Scheve 2013; Bechtel et al. 2017b).

We study the determinants of citizen preferences for sharing COVID-19 vaccines through a Conjoint design. In addition, we investigate whether information campaigns could increase international solidarity among respondents.

4.2 Design

The experiment relies on a randomized intervention followed by a conjoint experiment with factors assigned with independent probabilities. The unit of randomization for the video-treatment is the individual. For the conjoint experiment, each respondent will receive three vignettes successively. However, respondent will not see that same profile twice.

4.3 Hypotheses

Hypothesis 1: Information campaign We expect that the amount of money personally donated for global vaccine sharing is higher for respondents in the treatment as compared to the control group.

Hypothesis 2: Absolute contribution We expect that the willingness to share vaccines decreases with an increase in the number of vaccine doses that Germany should share.

Hypothesis 3: Relative contribution We expect that the willingness to share vaccines decreases with an increase in the contribution of vaccine doses that Germany should share in comparison to other countries.

Hypothesis 4: Economic benefits We expect that the willingness to share vaccines is higher if the receiving country is economically important to Germany.

Hypothesis 5: Health benefits We expect that the willingness to share vaccines is higher if the receiving country constitutes a risk of infection for Germany.

Hypothesis 6: Interaction effect Information campaign We expect that the information campaign video treatment has a positive interaction effects with all Conjoint attributes.

4.4 Exploratory Analysis

Additionally, we explore several heterogeneous treatment effects. In particular, we are interested to find out how preferences vary for different subgroups. We explore the interaction of the treatment arms with: age, partisanship, political ideology, European identity, attitudes towards EU integration, attitutdes towards immigration, altruism, reciprocity, religiosity, vaccination status and health conditions. We remain agnostic about the expected effect sizes. To explore heterogenous treatment effects, we also rely on machine learning algorithms like causal forests.

4.5 Randomized Intervention: Information campaign

We conduct a randomized intervention in which we randomly assign respondents to treatment (exposure to video) and control group.

- **Introductory text**: And now we come to the topic of the global vaccination campaign. The pandemic can only be defeated if it is brought under control globally. In the fight against Covid-19, the provision of vaccines is particularly important. The COVAX platform was set up under the leadership of the World Health Organization (WHO) for the acquisition and fair distribution of vaccines.

- **Video**: The video can be found here: https://www.dw.com/de/impfstoff-f%C3%BCr-entwicklungsl%C3%A4nder/av-56554104

4.6 Outcome: Willingness to share

4.7 Outcome: Personal donation

- **Personal donation**: You can also contribute to the global distribution of the vaccines yourself. UNICEF is working on behalf of the COVAX initiative to ensure that the corona vaccines are made available to people in the poorest countries.

  Next to the 75 Mingle points that you receive for taking part in this survey, you will receive an **additional 50 Mingle points** from us. You can either keep these points yourself or donate all or part of them to UNICEF for the worldwide distribution of corona vaccines. For every mingle point you donate, we donate 1.5 mingle points to UNICEF.

  Please select how the additional Mingle points should be allocated to you or UNICEF.

	Your Bonus	Donation to UNICEF
1	0 Mingle Points	75 Mingle Points
2	10 Mingle Points	60 Mingle Punkte
3	20 Mingle Points	45 Mingle Points
4	30 Mingle Points	30 Mingle Points
5	40 Mingle Points	15 Mingle Points
6	50 Mingle Points	0 Mingle Points

- *Donations go here:* https://www.unicef.de/spenden/jetzt-spenden?purpose=235762

4.8 Conjoint Design

- **Introductory text**: In the following, we present suggestions on how Germany's contribution to the global distribution of vaccine doses to poorer countries could look like this year.
- **Conjoint Design**: Our second outcome measure is a conjoint experiment (two profiles) with randomly assigned factors (uniform) over five attributes. Each respondents will see three pairs. Unit of randomization is respondent pair.

	Proposal
Sharing of vaccination doses	{0} Germany will give away 1 Million doses of its Covid Vaccine<br>{1} Germany will give away 5 Million doses of its Covid Vaccine<br>{2} Germany will give away 10 Million doses of its Covid Vaccine<br>{3} Germany will give away 20 Million doses of its Covid Vaccine
Germany's contribution compared to other countries	{0} Germany contributes 1% of the vaccines donated worldwide<br>{1} Germany contributes 5% of the vaccines donated worldwide<br>{2} Germany contributes 10% of the vaccines donated worldwide<br>{3} Germany contributes 20% of the vaccines donated worldwide
How many countries share vaccination doses	{0} 160 countries<br>{1} 80 countries<br>{2} 20 countries
Recipient countries - economic benefit	{0} Countries in need with economic importance for Germany<br>{1} Countries in need, even without economic importance for Germany
Recipient countries - risk of infection	{0} Countries in need from which there is a risk of infection for Germany<br>{1} Countries in need even if there is no risk of infection for Germany

4.9 Outcomes:

- **Forced Choice:** *Displayed as bottom row in the conjoint table*
  - Which proposal do you prefer? (*Respondents choose between both displayed options*)
- **Opposition to solidarity:** *The following tables is displayed below the conjoint table*

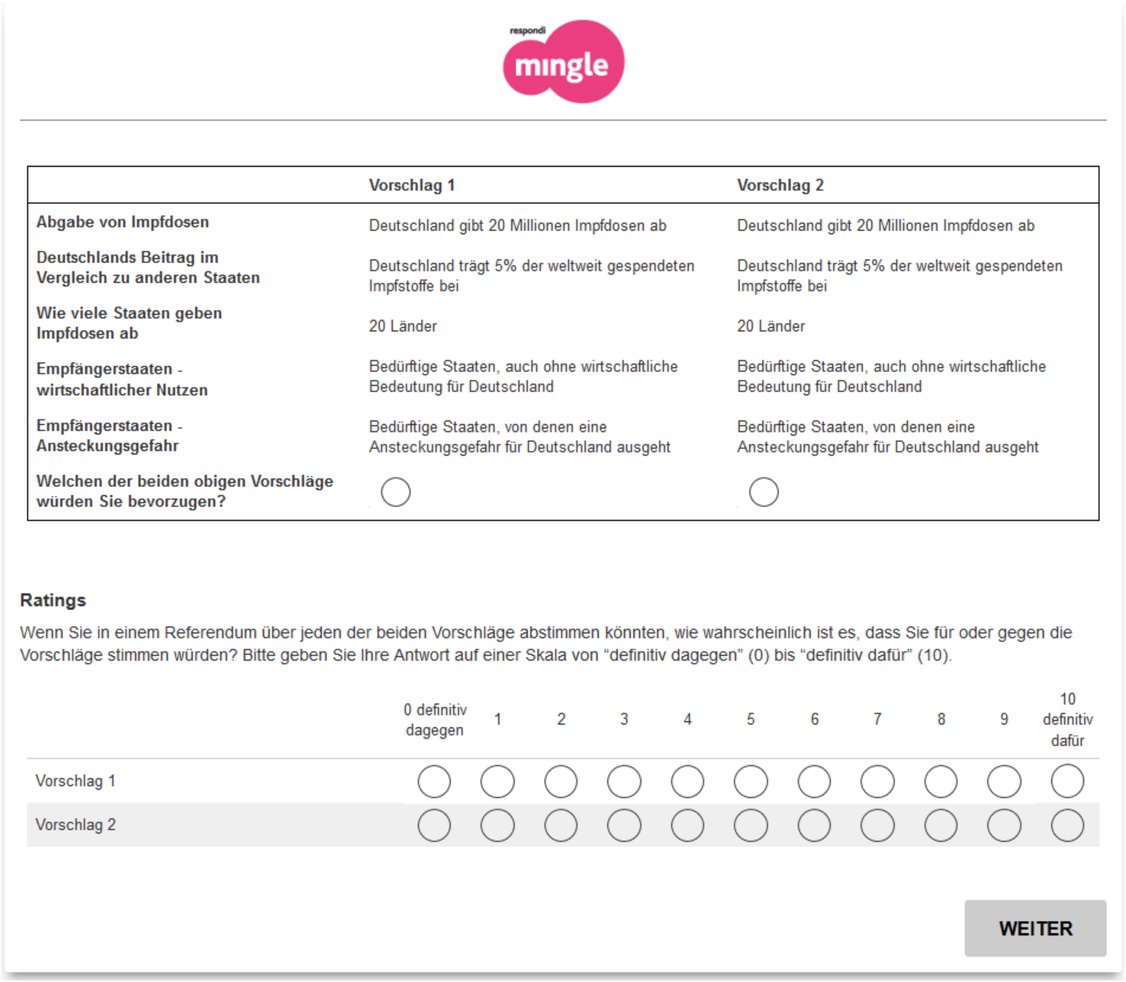

respondi mingle

	Vorschlag 1	Vorschlag 2
Abgabe von Impfdosen	Deutschland gibt 20 Millionen Impfdosen ab	Deutschland gibt 20 Millionen Impfdosen ab
Deutschlands Beitrag im Vergleich zu anderen Staaten	Deutschland trägt 5% der weltweit gespendeten Impfstoffe bei	Deutschland trägt 5% der weltweit gespendeten Impfstoffe bei
Wie viele Staaten geben Impfdosen ab	20 Länder	20 Länder
Empfängerstaaten - wirtschaftlicher Nutzen	Bedürftige Staaten, auch ohne wirtschaftliche Bedeutung für Deutschland	Bedürftige Staaten, auch ohne wirtschaftliche Bedeutung für Deutschland
Empfängerstaaten - Ansteckungsgefahr	Bedürftige Staaten, von denen eine Ansteckungsgefahr für Deutschland ausgeht	Bedürftige Staaten, von denen eine Ansteckungsgefahr für Deutschland ausgeht
Welchen der beiden obigen Vorschläge würden Sie bevorzugen?	○	○

Ratings

Wenn Sie in einem Referendum über jeden der beiden Vorschläge abstimmen könnten, wie wahrscheinlich ist es, dass Sie für oder gegen die Vorschläge stimmen würden? Bitte geben Sie Ihre Antwort auf einer Skala von "definitiv dagegen" (0) bis "definitiv dafür" (10).

	0 definitiv dagegen	1	2	3	4	5	6	7	8	9	10 definitiv dafür
Vorschlag 1	○	○	○	○	○	○	○	○	○	○	○
Vorschlag 2	○	○	○	○	○	○	○	○	○	○	○

WEITER

4.10 Estimands:

Our estimands are the ATE of the video treatment, the Average Marginal Component Effect (AMCE) of the randomly-assigned factors, and the conditional AMCE's depending on the video assignment. The AMCE is the marginal effect of each factor, averaged over the joint distribution of other attributes. The AMCE measures the average difference to which a given value of a profile feature increases or decreases respondents' support for the overall profile relative to a baseline, averaging across the joint distribution over other attributes.

4.11 Estimation:

First, we estimate the treatment effects using a OLS regression with heteroskedasticity-robust standard errors: $Y_{im} = \sum_{j \in Z} \beta_j Z_{ij} + \gamma_m + \epsilon_i$ where Y is the chosen profile, j indexes the treatment level, γ_m indicates individual fixed effects, and Z is a set of indicators corresponding to the attributes, here Z = {Costs, Burden, Participation, Economic Benefits, Health Benefits}. Second, we estimate the conditional AMCE with respect to the treatment variables T information video: $Y_{im} = \sum_{j \in Z} \beta_j Z_i^j + \sum_{j \in Z} \theta_j (Z_i^j * T_i) + \alpha T_i + \sum X_i + \gamma_m + \epsilon_i$ where θ is the conditional AMCEs. Third, we estimate the ATE of the video treatment on the donation outcome using a simple OLS: $Y_i = \beta_0 + \beta_1 T_i + \epsilon_i$ where Y is the solidarity outcome, β_0 estimates the average among control observations and β_1 estimates the differences of means between treatment and the control group.

[ ]:
```
%load_ext rpy2.ipython
```

[ ]:
```
%%R
# packages ------------------------------------------------------------
#install.packages("DeclareDesign")
#install.packages("tidyverse")
#install.packages("knitr")
#install.packages("stats")

library(DeclareDesign)
library(tidyverse)
library(knitr)
library(stats)
```

[ ]:
```
%%R
#############################################

# Ns
N_subjects <- 12500
N_pairs <- 3


# Direct treatment effect sizes (binary)
treatment_eff <- 0.1
economic_eff <- 0.1
health_eff <- 0.1

# Direct effect sizes (multi)
cost1_eff <- 0.05
cost2_eff <- 0.05
cost3_eff <- 0.05
burden1_eff <- 0.05
burden2_eff <- 0.05
burden3_eff <- 0.05
participation1_eff <- 0.05
participation2_eff <- 0.05


# Interactions
treatment_economic_interaction <- 0.05
treatment_health_interaction <- 0.05
treatment_cost_interaction1_eff <- 0.05
treatment_cost_interaction2_eff <- 0.05
treatment_cost_interaction3_eff <- 0.05
treatment_burden_interaction1_eff <- 0.05
treatment_burden_interaction2_eff <- 0.05
treatment_burden_interaction3_eff <- 0.05
```

```
treatment_participation_interaction1_eff <- 0.05
treatment_participation_interaction2_eff <- 0.05

pop <-declare_population(N = N_subjects * N_pairs,
                   pair = rep(1:(N / 2), each = 2),
                   noise = rnorm(N))


# Assign attributes independently
# binary
treatment <- declare_assignment(assignment_variable = "treatment", prob = .5)
economic <- declare_assignment(assignment_variable = "economic", prob = .5)
health <- declare_assignment(assignment_variable = "health", prob = .5)


# non binary
costs <- declare_assignment(assignment_variable = "costs",
                                       prob_each = c(1,1,1,1)/4,
                                       conditions = c("0", "1", "10","20"))


burden <- declare_assignment(assignment_variable = "burden",
                                        prob_each = c(1,1,1,1)/4,
                                        conditions = c("0", "5", "10","20"))

participation <- declare_assignment(assignment_variable = "participation",
                                  prob_each = c(1,1,1)/3,
                                  conditions = c("20", "80", "160"))

# choices

choice <-
  declare_step(
    # Gen utility
    utility = qnorm(
      0.0 +
        # Direct effects for binary attributes
        treatment * treatment_eff +
        economic * economic_eff +
        health * health_eff +
        # Direct effects for non binary attributes
        # leave baseline category out
        (costs == "1") * cost1_eff +
        (costs == "10") * cost2_eff +
        (costs == "20") * cost3_eff +
        (burden == "5") * burden1_eff +
        (burden == "10") * burden2_eff +
```

```
        (burden == "20") * burden3_eff +
        (participation == "80") * participation1_eff +
        (participation == "160") * participation2_eff +
        # Interaction binary attributes
        treatment * economic * treatment_economic_interaction +
        treatment * health * treatment_health_interaction +
        # Interaction between level and endorsement
        treatment * (costs == "1") * treatment_cost_interaction1_eff +
        treatment * (costs == "10") * treatment_cost_interaction2_eff
    ),
    # Generate binary support
    choice_binary = rbinom(n = N,size = 1,prob = pnorm(utility)),
    # Generate likert support (may not work out to pp effects)
    choice_likert = draw_ordered(N = N, x = rnorm(n = N,mean = pnorm(utility)),
 ↪breaks = c(-Inf, -1, 0, 1, Inf)),
    handler = fabricate
  )

# Define estimators  ----------------------------------------------------------

est_att_only <- declare_estimator(
  formula = choice_binary ~ economic + health,
  model = lm_robust,
  label = "Attributes only",
  term = TRUE)

est_type_cost <- declare_estimator(
  formula = choice_binary ~ costs,
  model = lm_robust,
  subset = costs %in% c("0", "1", "10","20"),
  label = "costs analysis",
  term = TRUE)


# binary interaction estimator
est_treatment_health <- declare_estimator(
  formula = choice_binary ~ treatment * health,
  model = lm_robust,
  label = "Interaction with treatment and health",
  term = TRUE)

# multi interaction estimator
est_treatment_costs_interaction <- declare_estimator(
  formula = choice_binary ~ treatment * I(costs == "20"),
  model = lm_robust,
  label = "Interaction with treatment and high level of costs",
  term = TRUE)
```

```
# Declare design ----------------------------------------------------------

design <-
  pop +
  # Assign binary attributes
  health + economic + treatment +
  # Assign multi attributes
  costs  + burden + participation+
  # Generate utilities and corresponding choices
  # Assign mediators
  choice +
  # Estimators
  est_att_only + est_type_cost +
  est_treatment_health  +est_treatment_costs_interaction

# Investigate design ------------------------------------------------------

# This is one draw of the estimates
draw_estimates(design)

# Do sims draws of estimates and get power by estimator and coefficient term
sims <- 100
simulations <- simulate_design(design, sims = sims)

# Manually calculate power or plot things using simulations dataframe
simulations %>%
  group_by(estimator_label, term) %>%
  summarize(power = mean(p.value <= .05))%>%
  kable("latex")

# Or use diagnose_design, which calculates power automatically
## Diagnosis
diagnosis <- diagnose_design(design, sims = sims)
power<-reshape_diagnosis(diagnosis, select = "Power")

power %>%
  kable("latex")
```

[ ]:
```
# Simulate and plot -------------------------------------------------------
%%R

ggplot(simulations, aes(estimate, fill = p.value <= 0.05)) +
  geom_histogram(bins = 30) +
  facet_wrap(~term, scales = "free_x") +
  theme_bw()
```

Design Label	Estimator Label	Term	N Sims	Power
design	Attributes only	(Intercept)	100	1.00
				(0.00)
design	Attributes only	economic	100	1.00
				(0.00)
design	Attributes only	health	100	1.00
				(0.00)
design	costs analysis	(Intercept)	100	1.00
				(0.00)
design	costs analysis	costs1	100	1.00
				(0.00)
design	costs analysis	costs10	100	1.00
				(0.00)
design	costs analysis	costs20	100	1.00
				(0.00)
design	Interaction with treatment and health	(Intercept)	100	1.00
				(0.00)
design	Interaction with treatment and health	health	100	1.00
				(0.00)
design	Interaction with treatment and health	treatment	100	1.00
				(0.00)
design	Interaction with treatment and health	treatment:health	100	1.00
				(0.00)
design	Interaction with treatment and high level of costs	(Intercept)	100	1.00
				(0.00)
design	Interaction with treatment and high level of costs	I(costs == "20")TRUE	100	0.69
				(0.05)
design	Interaction with treatment and high level of costs	treatment	100	1.00
				(0.00)
design	Interaction with treatment and high level of costs	treatment:I(costs == "20")TRUE	100	0.89
				(0.04)

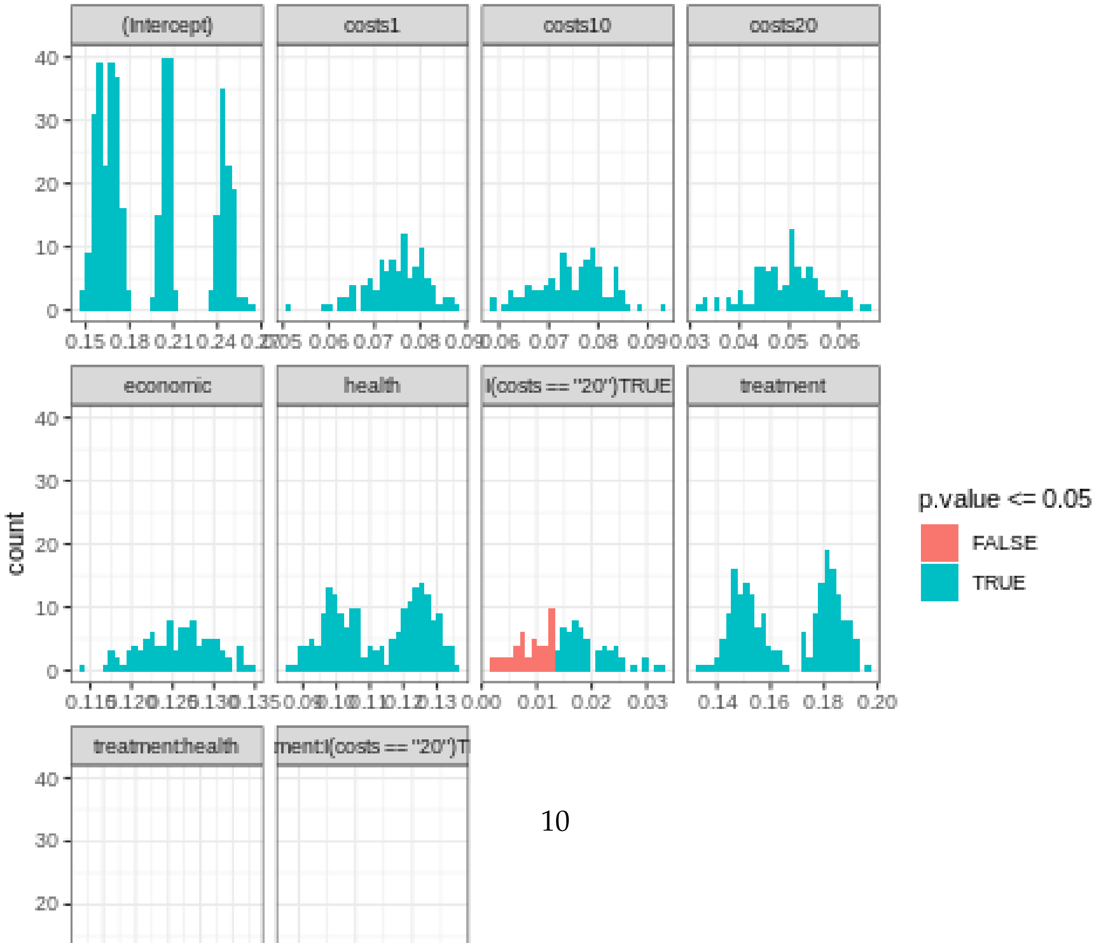

```
[ ]:
```
