# Supplementary material for: Public support for global vaccine sharing in the COVID-19 pandemic: Evidence from Germany
Source: PLoS One. 2022 Dec 14;17(12):e0278337. doi: 10.1371/journal.pone.0278337 (PMC9750013; doi:10.1371/journal.pone.0278337)
Supplement: S3 File — (PDF) [file pone.0278337.s003.pdf]

Supplementary Materials

Contents

A Sample & Recruitment **2**

B Results Refreshment Sample (Experiment 1) **2**

C Experiment Design **2**

C.1 Assignment . . . 3

C.2 Outcomes . . . 4

D Information Experiment Design **5**

D.1 Outcome: Willingness to share . . . 5

D.2 Outcome: Personal donation . . . 5

E Pre-registered subgroup analyses **6**

A Sample & Recruitment

Our population of interest consists of all German citizens aged 18 to 75 years. We fielded a multi-wave panel from which we use data from wave 2 and 4 in this study. In wave 1, the sample corresponded to the official national statistics with respect to age, sex and region ($N = 20,500$). Since we have attrition between the different waves, it is important to note that we do not have a representative sample of the German population (see Table 1). In wave 4 we implemented a refreshment sample which again corresponded to the official national statistics with respect to age, sex and region ($N = 2,215$).

For Experiment 2, we rely on a sample of $N = 13,782$ respondents from wave 2. For Experiment 4, we rely on a sample of $N = 10,525$ respondents from wave 4 which consists of $N = 8,400$ panel participants and $N = 2,215$ respondents from the refreshment sample. Experiment 2 was conducted from 29 April until 10 May 2021 and Experiment 1 from 8 until 22 September 2021. For recruitment, we relied on the online access panel of the survey company Respondi who uses online and offline channels to recruit new panelists for its online panel. Panelists receive a compensation from respondi for completing the survey. In wave 2, the incentive was EUR 0.95 for a median length of interview (LOI) of approximately 19 minutes. In wave 4, the incentive was EUR 0.95 for a median LOI of approximately 19 minutes for the panel respondents and EUR 1.15 for a median LOI of approximately 23 minutes for the refreshment sample.

B Results Refreshment Sample (Experiment 1)

For Experiment 1, our sample of $N = 10,525$ respondents consists of $N = 8,400$ panel participants who participated in all four waves and $N = 2,215$ respondents from the refreshment sample who were questioned for the first time. This gives us the opportunity to rerun the analyses separately for the panel participants and the freshly drawn random sample to check to what extent panel attrition might have impacted the results. Overall, the results look similar (see Fig 4) with the exception that for the refreshment sample we do not find a sizable effect for the amount given by other countries on the amount of cash or doses respondents are willing to contribute.

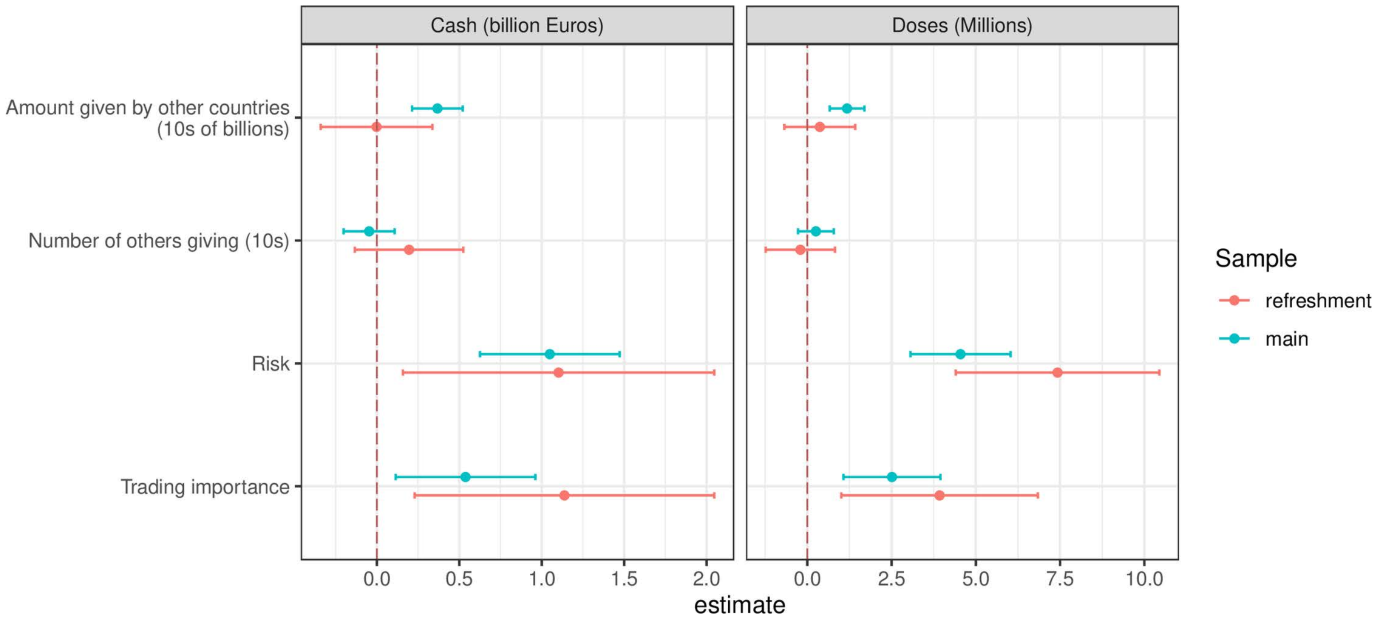

Fig 4. Levels of support by sample group

C Experiment Design

We implement a $2 \times 2 \times 5$ factorial design.

We lead with introductory text:

> The following is about vaccination progress in Germany and the rest of the world.
>
> The vaccination campaign against the coronavirus in Germany is now well advanced and anyone who wanted to be vaccinated could do so. The benefits of a third booster vaccination are currently being discussed. In contrast, many other, poorer countries are still at the very beginning with vaccinations and almost no one there has received a single vaccination yet.
>
> A total of around 11 billion vaccine doses are needed to vaccinate all people around the world who are ready to vaccinate. Although the production of

vaccines is in full swing, there is currently not enough vaccine available to carry out the first and second vaccinations in the poorer countries and the third vaccination in the richer countries at the same time

Variations are then as follows:

Z1: Trading importance

- 0 Control: It has no negative impact on the German economy if there are no vaccinations in poorer countries.
- 1 Treatment: The German economy shrinks by around 5% if there are no vaccinations in poorer countries.

Z2: Risk

- 0 Control: The risk of new mutations of the coronavirus does not increase noticeably in Germany if there are no vaccinations in poorer countries.
- 1 Treatment: The risk of new mutations of the coronavirus increases considerably in Germany if there are no vaccinations in poorer countries.

Z3: Deal

- 0 Control: There is no international deal on the global distribution of vaccines to poorer countries.
- 1 Treatment: There is an international deal on the global distribution of vaccines to poorer countries. 20 other countries are involved in the agreement, which together contribute a total of 20 billion euros.
- 2 Treatment: There is an international deal on the global distribution of vaccines to poorer countries. 40 other countries are involved in the agreement, which together contribute a total of 20 billion euros.
- 3 Treatment: There is an international deal on the global distribution of vaccines to poorer countries. 20 other countries are involved in the agreement, which together contribute a total of 40 billion euros.
- 4 There is an international deal on the global distribution of vaccines to poorer countries. 40 other countries are involved in the agreement, which together contribute a total of 40 billion euros.

Note that $Z3$ can itself be interpreted as a $2 * 2 + 1$ sub design that can be coded into:

- Z4: 0, 20, 40 other countries
- Z5: 0, 20, 40 billion provided by others

C.1 Assignment

Each subject sees two conditions, producing a total of $20 \times 19 = 380$ versions, assigned independently.

C.2 Outcomes

Subjects are asked about amounts of vaccines that Germany should share and financial contributions Germany should make in each condition.

Outcome 1:

> The total costs to meet global vaccination needs amount to around 70 billion euros. A contribution from Germany of one billion euros to this fund would cost the population in Germany the equivalent of around 12 euros per person.
>
> For each of the two scenarios, please indicate how many euros Germany should contribute to this fund for global vaccination. (Options range from 0 to 70 billion; Millions can be specified, separated by commas (e.g. 0.1 billion for 100 million))

- Vignette 1 ..billion Euro
- Vignette 2 ..billion Euro

Outcome 2:

> Germany will still have around 200 million vaccine doses available by the end of the year. If Germany wanted to offer all of its citizens a third vaccination, Germany would have to reserve around 70 million of these vaccine doses.
>
> For each of the two scenarios, please indicate what proportion of these 200 million vaccine doses Germany should contribute to the global distribution of vaccine doses to poorer countries. (Options range from 0 to 200m)

- Vignette 1 ..million doses
- Vignette 2 ..million doses

See Fig 5 for a screenshot of the experiment, as seen by respondents.

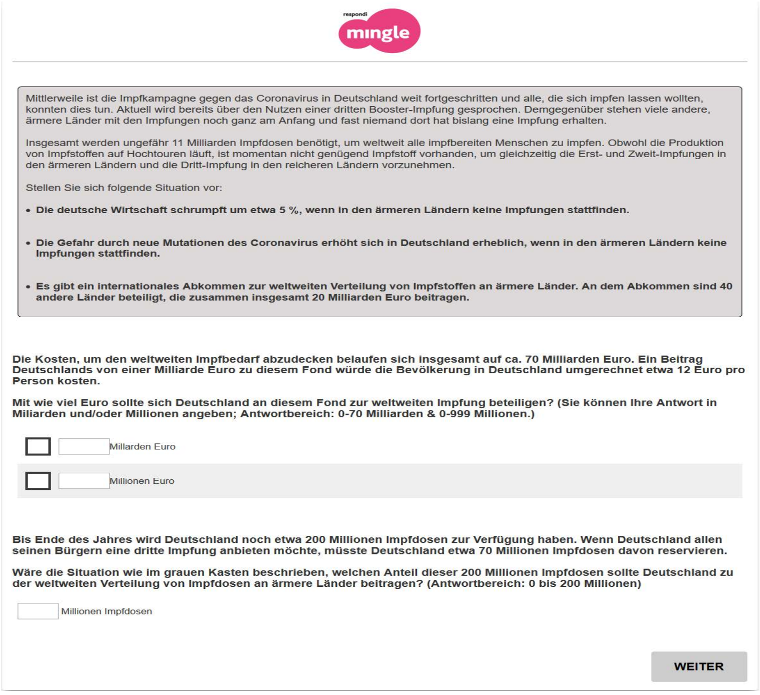
respondi
mingle

Mittlerweile ist die Impfkampagne gegen das Coronavirus in Deutschland weit fortgeschritten und alle, die sich impfen lassen wollten, konnten dies tun. Aktuell wird bereits über den Nutzen einer dritten Booster-Impfung gesprochen. Demgegenüber stehen viele andere, ärmere Länder mit den Impfungen noch ganz am Anfang und fast niemand dort hat bislang eine Impfung erhalten.

Insgesamt werden ungefähr 11 Milliarden Impfdosen benötigt, um weltweit alle impfbereiten Menschen zu impfen. Obwohl die Produktion von Impfstoffen auf Hochtouren läuft, ist momentan nicht genügend Impfstoff vorhanden, um gleichzeitig die Erst- und Zweit-Impfungen in den ärmeren Ländern und die Dritt-Impfung in den reicheren Ländern vorzunehmen.

Stellen Sie sich folgende Situation vor:

- **Die deutsche Wirtschaft schrumpft um etwa 5 %, wenn in den ärmeren Ländern keine Impfungen stattfinden.**
- **Die Gefahr durch neue Mutationen des Coronavirus erhöht sich in Deutschland erheblich, wenn in den ärmeren Ländern keine Impfungen stattfinden.**
- **Es gibt ein internationales Abkommen zur weltweiten Verteilung von Impfstoffen an ärmere Länder. An dem Abkommen sind 40 andere Länder beteiligt, die zusammen insgesamt 20 Milliarden Euro beitragen.**

Die Kosten, um den weltweiten Impfbedarf abzudecken belaufen sich insgesamt auf ca. 70 Milliarden Euro. Ein Beitrag Deutschlands von einer Milliarde Euro zu diesem Fond würde die Bevölkerung in Deutschland umgerechnet etwa 12 Euro pro Person kosten.

Mit wie viel Euro sollte sich Deutschland an diesem Fond zur weltweiten Impfung beteiligen? (Sie können Ihre Antwort in Miliarden und/oder Millionen angeben; Antwortbereich: 0-70 Milliarden & 0-999 Millionen.)

Millarden Euro

Millionen Euro

Bis Ende des Jahres wird Deutschland noch etwa 200 Millionen Impfdosen zur Verfügung haben. Wenn Deutschland allen seinen Bürgern eine dritte Impfung anbieten möchte, müsste Deutschland etwa 70 Millionen Impfdosen davon reservieren.

Wäre die Situation wie im grauen Kasten beschrieben, welchen Anteil dieser 200 Millionen Impfdosen sollte Deutschland zu der weltweiten Verteilung von Impfdosen an ärmere Länder beitragen? (Antwortbereich: 0 bis 200 Millionen)

Millionen Impfdosen

WEITER

Fig 5. Screenshot of the interface

D Information Experiment Design

The information experiment was implemented in Wave 2. The experiment was introduced with the following text:

- **Introductory text**: And now we come to the topic of the global vaccination campaign. The pandemic can only be defeated if it is brought under control globally. In the fight against Covid-19, the provision of vaccines is particularly important. The COVAX platform was set up under the leadership of the World Health Organization (WHO) for the acquisition and fair distribution of vaccines.

The treatment, assigned to half of the participants, was a video produced by Deutsche Welle. Deutsche Welle is Germany's foreign broadcaster which is organized under public law and financed by federal tax revenues. The video can be viewed here: `https://www.dw.com/de/impfstoff-f%C3%BCr-entwicklungsl%C3%A4nder/av-56554104`

D.1 Outcome: Willingness to share

Attitudinal outcome: Would you be willing to personally support the international distribution of vaccines?

1. Yes
2. No

D.2 Outcome: Personal donation

- **Personal donation**: You can also contribute to the global distribution of the vaccines yourself. UNICEF is working on behalf of the COVAX initiative to ensure that the corona vaccines are made available to people in the poorest countries.

  Next to the 75 Mingle points that you receive for taking part in this survey, you will receive an **additional 50 Mingle points** from us. You can either keep these points yourself or donate all or part of them to UNICEF for the worldwide distribution of corona vaccines. For every mingle point you donate, we donate 1.5 mingle points to UNICEF.

  Please select how the additional Mingle points should be allocated to you or UNICEF.

Table 4. Bonuses and donations

	Your Bonus	Donation to UNICEF
1	0 Mingle Points	75 Mingle Points
2	10 Mingle Points	60 Mingle Points
3	20 Mingle Points	45 Mingle Points
4	30 Mingle Points	30 Mingle Points
5	40 Mingle Points	15 Mingle Points
6	50 Mingle Points	0 Mingle Points

- *Donations go here:* `https://www.unicef.de/spenden/jetzt-spenden?purpose=235762`

E Pre-registered subgroup analyses

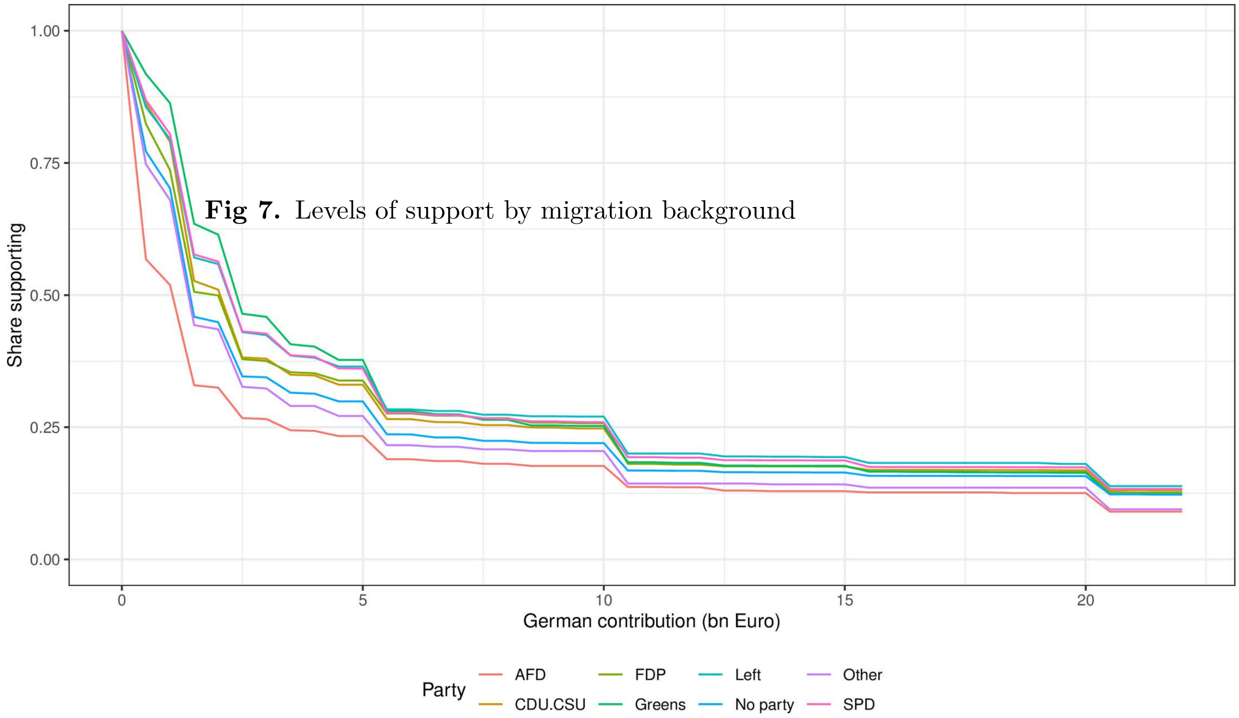

Fig 7. Levels of support by migration background

Fig 6. Levels of support by party

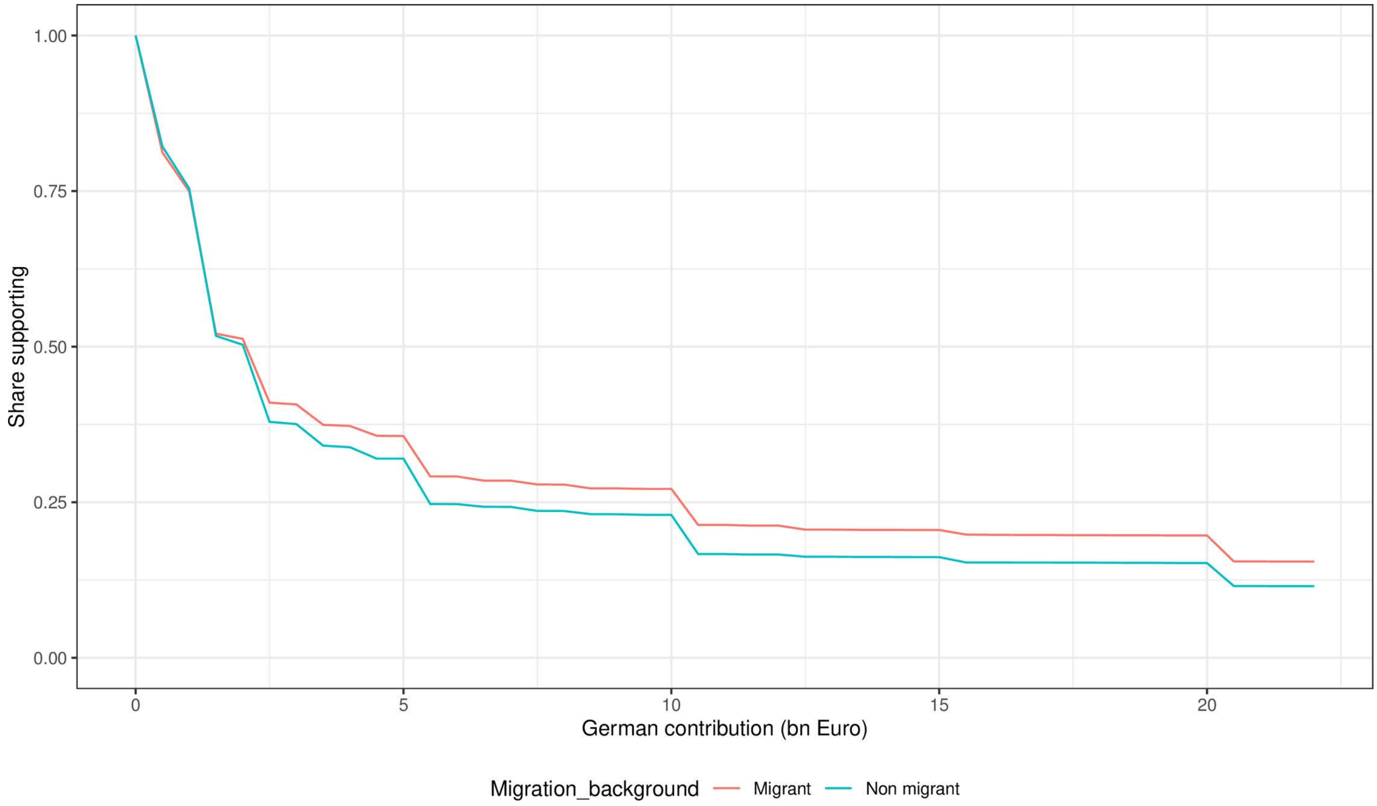

Fig 7. Levels of support by migration background

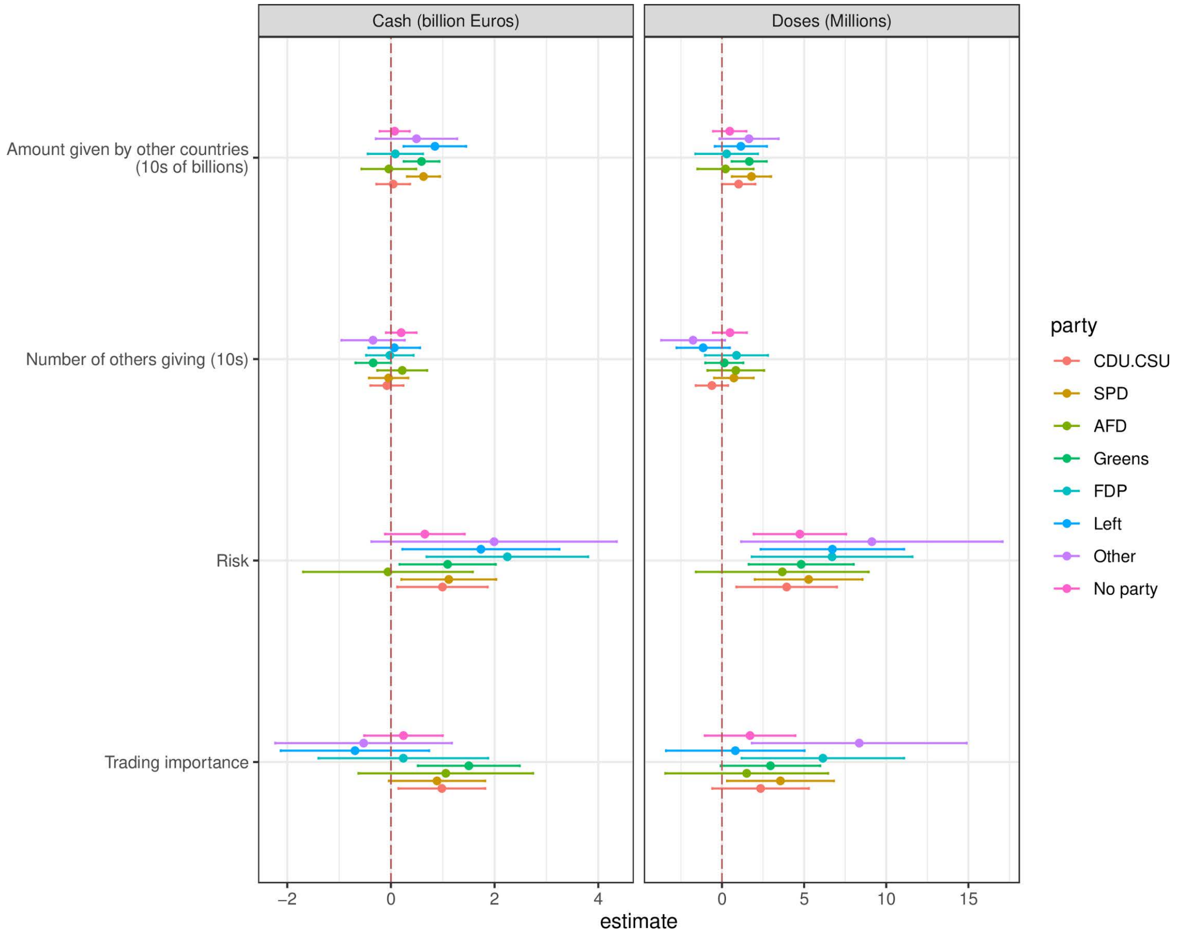

Fig 8. Marginal effects of conditions by party

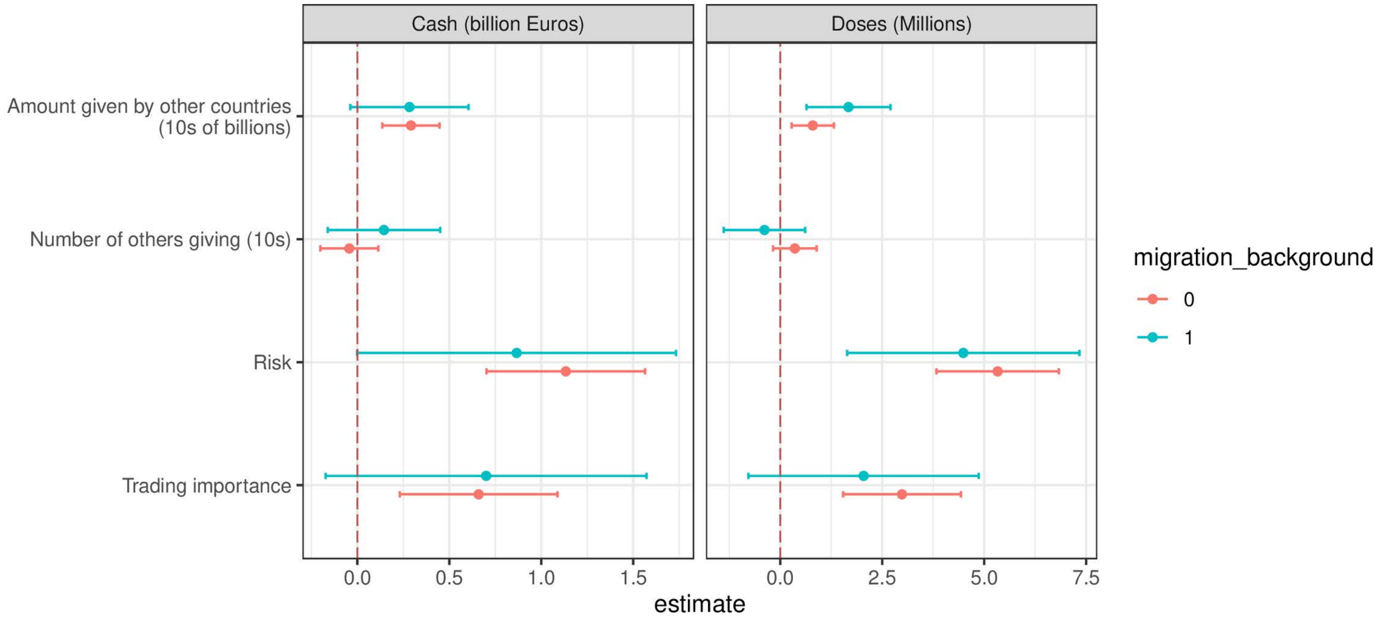

Fig 9. Marginal effects of conditions by migration background
