# Supplementary material for: Public support for global vaccine sharing in the COVID-19 pandemic: Evidence from Germany
Source: PLoS One. 2022 Dec 14;17(12):e0278337. doi: 10.1371/journal.pone.0278337 (PMC9750013; doi:10.1371/journal.pone.0278337)
Supplement: Attachment — Submitted filename: Response_to_Reviewers.pdf [file pone.0278337.s004.pdf]

Dear Reviewers,

Thank you again or the thoughtful reviews of our manuscript. We have endeavored to address all of your concerns and suggestions.

Below, we explain in detail the modifications we have made. Reviewer comments are written in italics; our responses follow in normal font. To indicate changes that we have conducted in the manuscript, we highlight all changes in the main text in red.

Comments to the Author

1. If the authors have adequately addressed your comments raised in a previous round of review and you feel that this manuscript is now acceptable for publication, you may indicate that here to bypass the “Comments to the Author” section, enter your conflict of interest statement in the “Confidential to Editor” section, and submit your "Accept" recommendation.

Reviewer #1: (No Response)

Reviewer #2: All comments have been addressed

---

2. Is the manuscript technically sound, and do the data support the conclusions?

The manuscript must describe a technically sound piece of scientific research with data that supports the conclusions. Experiments must have been conducted rigorously, with appropriate controls, replication, and sample sizes. The conclusions must be drawn appropriately based on the data presented.

Reviewer #1: Yes

Reviewer #2: Yes

---

3. Has the statistical analysis been performed appropriately and rigorously?

Reviewer #1: Yes

Reviewer #2: Yes

---

4. Have the authors made all data underlying the findings in their manuscript fully available?

The PLOS Data policy requires authors to make all data underlying the findings described in their manuscript fully available without restriction, with rare exception (please refer to the Data Availability Statement in the manuscript PDF file). The data should be provided as part of the manuscript or its supporting information, or deposited to a public repository. For example, in addition to summary statistics, the data points behind means, medians and variance measures should be available. If there are restrictions on publicly sharing data—e.g. participant privacy or use of data from a third party—those must be specified.

Reviewer #1: Yes

Reviewer #2: Yes

---

5. Is the manuscript presented in an intelligible fashion and written in standard English?

PLOS ONE does not copyedit accepted manuscripts, so the language in submitted articles must be clear, correct, and unambiguous. Any typographical or grammatical errors should be corrected at revision, so please note any specific errors here.

Reviewer #1: Yes

Reviewer #2: Yes

---

6. Review Comments to the Author

Please use the space provided to explain your answers to the questions above. You may also include additional comments for the author, including concerns about dual publication, research ethics, or publication ethics. (Please upload your review as an attachment if it exceeds 20,000 characters)

Reviewer #1: The authors have been largely responsive to my initial comments. A few minor comments remain:

1- Abstract:

** Information in the abstract does not match the Introduction; please update the abstract (50% of the global population is vaccinated, etc., 85% vaccination in top 10 countries, etc.).*

- We thank the reviewer for pointing to this inconsistency. We have updated all numbers to match the timing of the survey. Though this is not the most recent data it is the most relevant for interpreting results and avoids working with a moving target.

2- Introduction:

** Factual data needs citation (number of doses administered globally, etc.).*

- Thank you. We write now in a footnote:

  “Numbers calculated based on data provided by Mathieu (2021); calculations available in replication materials. Top 10 and bottom 10 countries are from among 102 countries with populations > 500,000 that report vaccination rates.”

  We also now more clearly indicate the relevant section of the replication file (it is now labeled “3. Numbers provided in Introduction (Vaccine background data from OWID)”) and all calculations are implemented more transparently with consistent dates.

** I suggest you change "disease mutation" to "emergence of SARS-CoV-2 variants/mutations".*

- We agree that this is more accurate and have changed it accordingly.

** Vaccines are not highly effective in preventing transmission of Omicron variants, but they still prevent morbidity and mortality associated with the disease. This should be noted in the 2nd paragraph.*

- We reformulated the sentence as follows to capture this as well:

  “The rise of the Omicron variant cannot be stopped with the vaccines that are currently available, but at least the spread can be slowed down and morbidity and mortality associated with the disease can be reduced..”

** Several countries have recommended a fourth dose for some populations, not only Israel (US, Brazil, etc.). Please update.*

- That is correct. In the meantime more countries followed with this recommendation. We have adjusted this in the text.

  “Despite the importance of globally distributing COVID-19 vaccines to stop the pandemic, most Western countries have launched campaigns for a third shot and a number of countries even started the distribution of a fourth vaccination (e.g. Israel, USA, Chile, Denmark or Germany) in the wake of the Omicron wave (Mahase, 2021).”

3- Methods

** I believe there is a confusion regarding the meaning of "nationally representative sample". Having the same proportion of men/women, or the same age distribution of the general population does not necessarily mean that the sample is nationally representative. Representativeness of the sample is more about the sampling strategy and if everyone in the target population could have been in the study. Is that the case in this study? Is this a random sample of the German population? Is this a systematic sampling approach?*

- We agree with the reviewer. We have reformulated this part making it even more clear that the sample is not a "nationally representative sample", though we also detail how on available observable characteristics of the sample match the population. We also reformulated the paragraph in the Supplementary Materials A. accordingly

  “Our study draws on two survey experiments that we fielded in a multi-wave panel study in Germany using the online access panel of the survey company Respondi. Respondi relies on online channels and offline channels to recruit new panelists for its online panel. After completing a profiling questionnaire covering basic sociodemographic information, panelists are then invited to participate in surveys. Respondi compensates its panelists for completing a survey.

  Our target population consists of all German citizens aged 18 to 75 years. In wave 1, the sample corresponded closely to the official national statistics with respect to age, sex and region though the quality of this correspondence weakened somewhat by

wave 4 (for details, see Table 1 and Section A in the Supplementary Materials). We conducted the experiments in wave 2 and 4 of the panel. All analyses were specified in preregistered analysis plans and the study obtained IRB approval at [Redacted Institution]. Data and replication material are publicly available at https://wzb-ipi.github.io/vaccine_solidarity/".

** Data access: I could not access the data file through the weblink provided, only the code used in analyses. Perhaps I am missing something obvious here, but I suspect other readers may also have trouble accessing the data used in this report.*

- The data will be made available under the provided weblink as soon as the paper gets published.
- We have edited the replication code however so that it runs from the cleaned data which we have made available already. As of now the reviewer should be able to replicate all analyses from the github replication archive.

** If possible, move Supplemental Table A to the main text, as we know that many readers do not access suppl materials.*

- Following this suggestion, we moved the Table into the main text.

**There should be a section in the Methods that describes the analytical approach employed.*

- We have adopted this suggestion and added a new section on analytic procedures/

4- Results

** I still think that the specification of the models should be discussed/explained in the Methods.*

- We have adopted this suggestion and added a new section on analytic procedures/

** I disagree that effect sizes are small. Take, for example, the effect of risk, which leads to a billion euro increase in donations (50% of the median contribution). This may be small compared to Germany's overall budget, but it seems like a substantial effect using the median donations as a benchmark. The responsiveness to health risks is also aligned with the significant effect of the educational video in increasing donations in Experiment 2.*

- We agree that the size of the effects could be evaluated differently depending on the benchmark. Therefore we reformulated the sentence.

  "Overall we see that both health risks and trading importance are statistically distinguishable from zero. While this effect may be small compared to Germany's overall budget, it is a sizable effect when using the median donations as a benchmark.

** It is unclear to me why authors used the dichotomous outcome (donate all to UNICEF vs. not) rather than the continuous amount of Mingle Points donated. Please clarify.*

- We do not use the dichotomous outcome in the analysis, we use the full data. In fact much of the data is in the all or nothing categories (see replication material section 8). This note on the differences in the highest category was intended to provide intuition for the movement behind the estimate which largely reflects this increase in the highest category. We have removed this point however to avoid confusion and simply note the average effect.

5- Discussion

** The authors continue to present new findings in the Discussion. These should be moved to the Results (and included in the Methods) or excluded from this report.*

Page 6: "we furthermore included a question asking respondents to indicate how the German government should prioritize to ensure vaccination for an older Indian woman as compared to a younger German woman in the second and fourth wave of the panel study.The comparison directly pits nationalist concerns against humanitarian concerns. Importantly also, we expect, any strategic considerations that enter in the decision to prioritize an Indian woman should apply a fortiori to the prioritization of a German woman. Strikingly, we nd that 57% of the respondents place the priority of the older Indian woman as high or higher than the German woman, and 38% even place it strictly higher."

- We moved this paragraph to the Results section.

** Were participants made aware of the cost of vaccines? The mismatch between money vs. vaccine contributions may be due to participants not knowing the average cost of vaccine doses.*

- We did not tell the respondents directly about the average costs of vaccine doses. However, we stated that around 11 billion vaccine doses would be needed globally and that the costs to cover these would be around 70 billion (see Figure 5). So the respondents had an idea about the actual costs of one vaccine dose.

Reviewer #2: (No Response)
